# Structural Basis of Targeting the Exportin CRM1 in Cancer

**DOI:** 10.3390/cells4030538

**Published:** 2015-09-18

**Authors:** Achim Dickmanns, Thomas Monecke, Ralf Ficner

**Affiliations:** Abteilung für Molekulare Strukturbiologie, Institut für Mikrobiologie und Genetik, GZMB, Georg-August-Universität Göttingen, Justus-von-Liebig-Weg 11, Göttingen 37077, Germany; E-Mails: tmoneck@uni-goettingen.de (T.M.); rficner@uni-goettingen.de (R.F.)

**Keywords:** XPO1, exportin1, nuclear transport, NES, cancer, CRM1, drugs, inhibitor

## Abstract

Recent studies have demonstrated the interference of nucleocytoplasmic trafficking with the establishment and maintenance of various cancers. Nucleocytoplasmic transport is highly regulated and coordinated, involving different nuclear transport factors or receptors, importins and exportins, that mediate cargo transport from the cytoplasm into the nucleus or the other way round, respectively. The exportin CRM1 (Chromosome region maintenance 1) exports a plethora of different protein cargoes and ribonucleoprotein complexes. Structural and biochemical analyses have enabled the deduction of individual steps of the CRM1 transport cycle. In addition, CRM1 turned out to be a valid target for anticancer drugs as it exports numerous proto-oncoproteins and tumor suppressors. Clearly, detailed understanding of the flexibility, regulatory features and cooperative binding properties of CRM1 for Ran and cargo is a prerequisite for the design of highly effective drugs. The first compound found to inhibit CRM1-dependent nuclear export was the natural drug Leptomycin B (LMB), which blocks export by competitively interacting with a highly conserved cleft on CRM1 required for nuclear export signal recognition. Clinical studies revealed serious side effects of LMB, leading to a search for alternative natural and synthetic drugs and hence a multitude of novel therapeutics. The present review examines recent progress in understanding the binding mode of natural and synthetic compounds and their inhibitory effects.

## 1. Introduction

In the early days of cancer treatment, shotgun approaches using drugs interfering with DNA replication in a more general way were used, with the consequence of massive unwanted side effects. About 30 years ago, identification of the individual proteins involved in specific cancers and an understanding of their biochemistry incited hype about having found the cure for cancer. In subsequent years, inhibitors identified to block these proteins allowed specific treatment of cancers, but the problem of resistances arose concurrently. At present, a multitude of proteins interfering with cell regulation have been described, but the specific amount of proteins required/involved to trigger cell cancerogenesis and the specific functions including the complex interplay of these proteins is still poorly understood. Recent understanding of interactions in the intermingled cellular pathways revived the discussion about “the” cure for cancer or the need for a more personalized and cancer-specific treatment targeting the individual deregulating mechanisms in each patient.

Recent developments in cancer therapy reveal that the effects of specific drugs may be increased by interfering with additional macromolecular machineries in the cell [1]. Due to the differences in cell metabolism between normal and cancer cells, such machineries are more stringently required by the latter. One example is the transport machinery, which is required for exchange of proteins and RNAs between the nuclear and the cytoplasmic compartment in all eukaryotic cells. This review focuses on CRM1-dependent export deregulation and effects of drugs on CRM1 function. For further reading as introduction to the complexity of cancer development, regulation and treatment, we refer to excellent reviews and perspective articles [1,2,3,4,5].

## 2. Observation: Alteration of Distribution of Proteins Related to Cancer

In cancerogenic cells, tumor suppressor proteins and oncoproteins are often aberrantly mislocalized. Mislocalization may be either due to any kind of deregulation of the protein biosynthesis pathway, malfunctioning of the protein itself or aberrations in the transport processes that are required to shuffle proteins from the cytoplasmic compartment into the nuclear compartment and *vice versa*. The malfunctioning of the latter process may result in a deregulation either by inactivation or by over-activation of the critical proteins for cell cycle regulation or growth and division. Such pathway-specific deregulation may cause an overall deregulation of the cell, ultimately leading to the establishment of cancer.

**Figure 1:**
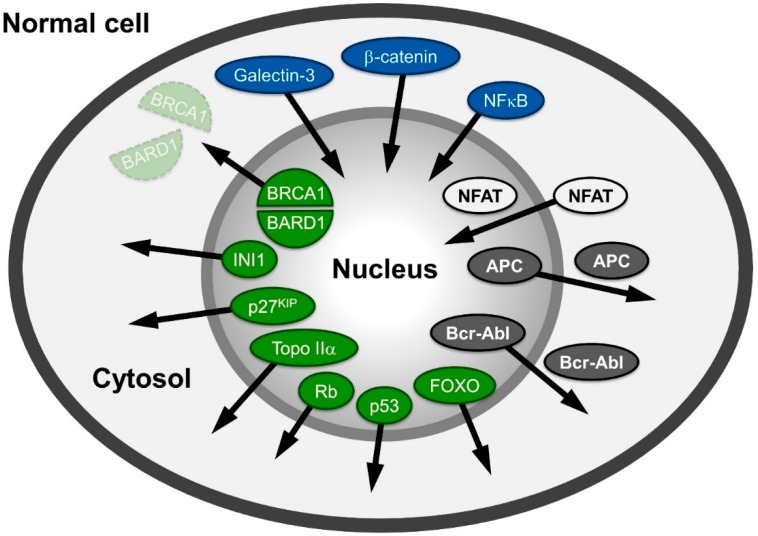
Spatial relocalization of (proto-) oncoproteins in cancer cells compared to normal cells. In cancer cells, proteins mislocalized into the nucleus are depicted in blue; in the cytoplasm, they are colored green; and those shifting from nuclear and cytoplasmic distribution to either cytoplasmic or nuclear are depicted in grey and white, respectively. The arrows indicate the direction of the shift in cancer cells. The function of the proteins depicted here is described in the main text.

A large variety of proteins involved in human cancers, like APC (adenomatous polyposis coli protein), NFAT (nuclear factor of activated T-cells), β-catenin or Survivin, Rb (retinoblastoma protein), p53 and Bcr-Abl mislocalize in different cancer cells (Figure 1) and are reviewed in: [6,7,8]. The proteins mentioned are also representative examples of the different possibilities of distributional changes that could occur within a cell.

The tumor suppressor protein/transcription factor p53, named according to its apparent molecular weight, is localized in the nucleus in normal cells. It is often inactivated in cancer cells due to mutations leading to a “loss of function” (e.g., in its DNA-binding capabilities) or p53 is delocalized into the cytoplasm due to NES unmasking and active export [9,10] reviewed in [11,12]. Similarly, the tumor suppressor retinoblastoma protein (Rb) is localized in the nucleus in normal cells, but has been shown to be delocalized to the cytoplasm in specific cancers [13,14,15,16].

Another group of proteins populates both compartments in normal cells, but is shifted towards or excluded from one compartment in cancer cells. For example, the tumor suppressor APC regulates many cellular functions and in complex with two other proteins (Glycogen Synthase Kinase (GSK)-3β and Axin) promotes the degradation of β-catenin in the cytosol. In non-transformed cells, APC is found in the nucleus and the cytoplasm, albeit with a more pronounced accumulation in the cytoplasm, as it bears both import and export signals [17,18]. The mutated forms of APC, present in more than 60% of all colon cancer patients [19] are most commonly *C*-terminally truncated versions. Such truncations are incapable of binding to Axin and to accumulate in the nucleus, as the mediating residues are located in the *C*-terminal region. Thus, regulation of the proto-oncogene β-catenin (cadherin-associated protein), a key mediator of the canonical wnt signaling pathway, is lost. Instead of phosphorylation by GSK-3β and proteasome-dependent cytoplasmic degradation, it exhibits an increased import in many cancer cells and accumulates in the nucleus. β-catenin’s structural properties resembling those of nuclear transport receptors strengthen recent evidence suggesting a transport receptor-independent nuclear accumulation. Interestingly, β-catenin functions as a moonlighting (dual function) protein involved in regulation of cell-cell adhesion as well as gene transcription. Its binding to transcription factors causes gene transactivation and leads to tumor formation; reviewed in: [20,21].

The transcription factor family NFAT (nuclear factor of activated T-cells) is also found in both cellular compartments in normal cells. The import of NFAT is dependent on calcineurin, a serine/threonine phosphatase. Upon dephosphorylation in NFATs serine-rich region (SRR), a nuclear localization signal (NLS) is exposed and import can occur. Export of NFAT is stimulated by PKA and, interestingly, by the nuclear fraction of GSK-3β. In human solid tumors and hematological malignancies, isoforms of NFAT are constitutively activated and/or overexpressed, leading to increased nuclear accumulation and activation of the downstream targets reviewed in: [22,23,24,25,26].

An example of the accumulation of a protein distributed between both compartments in normal cells, but enriched in the cytoplasm in cancer cells, is the proto oncogene Bcr-Abl, a ~200 kDa protein resulting from a fusion of parts of the ABL1 (Abelson Murine Leukemia Viral Oncogene Homolog 1) and BCR (breakpoint cluster region) genes. The transcript of the Bcr-Abl gene fusion is functional as an abnormal kinase and stimulates proliferation of myeloid cells into chronic myelogenous leukemia cells [27]; reviewed in: [28,29]

The underlying mechanism for nucleocytoplasmic exchange of all of these proteins mentioned above requires soluble transport receptors that specifically recognize their cargoes by signals and transport them to the opposing compartment. The most versatile export factor CRM1 is required for the export of a plethora of proteins e.g., Rb [13] or the proto-oncogene p53 [9]. Their localization shift towards the cytoplasmic compartment is often an important prerequisite to stabilize the deregulation of the tumor cell and enable uncontrolled/permanent cell proliferation.

## 3. The Nucleocytoplasmic Transport Machinery

The interchange of metabolites between the nuclear and cytoplasmic compartment occurs by passive diffusion. In contrast, an active, receptor-mediated transport is required for proteins to enter the nucleus in order to regulate and transcribe DNA or for transport of RNA (-protein complexes) into the cytoplasm. Furthermore, certain proteins that have to perform their function only at specific time points during the cell cycle are therefore imported or exported in a highly regulated manner.

The site of transfer is the nuclear pore complex (NPC), a large supramolecular complex composed of more than 30 different proteins, the nucleoporins. They assemble into the structural framework of the NPC and form the meshwork gating the central aqueous channel of the NPC [30,31,32]. While this meshwork is no hindrance for small proteins and metabolites, large molecules require specific receptors for transition [30,31,32]. The receptors are classified in importins and exportins depending on their direction of transport with the nucleus as reference point. Many of them share structural properties to the first receptor identified, Importin-β (Impβ). Proteins of the Impβ superfamily of transport receptors are all composed of a common structural motif, the HEAT repeats named after the first proteins identified bearing this motif, namely Huntingtin, Elongation factor 3 (EF3), Protein Phosphatase 2A and the yeast P3 kinase Tor (Target of rapamycin). A single HEAT repeat covers 40–50 residues and is composed of two antiparallel α-helices, which are connected by a short linker loop [33,34]. The slight angular shift between the two helices and the overall stacking of the HEATs results in a superhelical protein conformation with a high intrinsic flexibility.

Directionality of transport depends on the small GTPase Ran that comes in two types, either in a GTP or GDP bound state, which are asymmetrically distributed in the nucleus and the cytoplasm [35]. In the nucleus, the Ran Guanine nucleotide Exchange Factor (RanGEF) RCC1 (Regulator of Chromosome Condensation 1) is bound to chromatin [36,37,38] and ensures a high nuclear concentration of RanGTP [39,40]. In the cytoplasm, the Ran-GTPase Activating Protein (RanGAP) and stimulatory factors Ran Binding Proteins 1 and 2 (RanBPs 1/2) are located, thereby resulting in high concentrations of RanGDP compared to RanGTP [41,42,43]. Interestingly, the eukaryotic translation initiation factor eIF4E, a potent oncogene, is not only involved in translation of bulk mRNA but additionally plays a role in the CRM1-mediated export of a subset of oncogene mRNAs. It was shown that eIF4E overexpression and dysregulation increases RanBP1 levels and reduces the amount of RanBP2. This leads to a faster and more efficient export of the eIF4E/CRM1-dependent mRNAs and their subsequent translation and thus an increased oncogenic potential [44,45].

**Figure 2:**
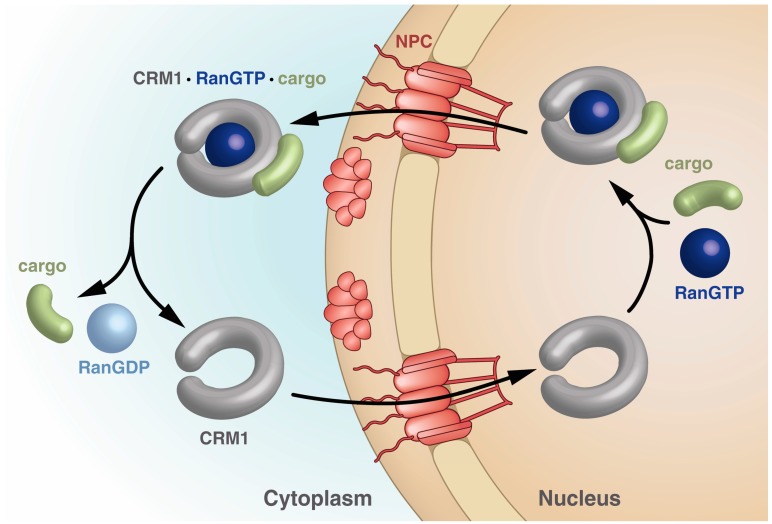
Schematic drawing of the steps within the CRM1 transport cycle. The steps depicted highlight the different states of CRM1 with respect to the overall shape as well as points of cargo and RanGTP binding and release. See text for details.

Importins bind their cargo in the cytoplasm and release it upon binding of RanGTP in the nucleus, while exportins bind cargo in the nucleus only in the presence of RanGTP and release it in the cytoplasm upon Ran-driven GTP hydrolysis stimulated by RanBPs and RanGAP. The export receptor returns empty into the nucleus for another round of export (Figure 2).

### 3.1. Bi-Functional CRM1: Discovery as an Export Receptor and Cell Cycle Control Factor

CRM1 has first been identified in a cold-sensitive strain of the budding yeast *Schizosaccharomyces pombe* where its mutation causes abnormal chromosome morphology at restrictive temperatures [46]. Later, CRM1 was shown to interact with Can/Nup214 [47,48], a protein located at the cytoplasmic side of the nuclear pore complex (NPC).

Since then, *in vitro* and *in vivo* experiments clearly demonstrated the role of CRM1 as a major nuclear export receptor [49,50,51,52,53,54] and identified its cargoes as proteins, which carry a leucine-rich—classical—nuclear export signal (NES). The first NESes were identified in the human immunodeficiency virus type 1 (HIV-1) protein Rev (regulator of expression of virion proteins) and in the cellular protein kinase A inhibitor PKI [55,56,57,58]. More complex export events, like the export of m^7^G-capped snRNAs may require additional proteins: e.g., the Cap Binding Complex (CBC; consisting of the two cap binding proteins 20 and 80) in addition to PHAX (phosphorylated adaptor of RNA export), which provides the NES [59,60]. In fact, the aforementioned HIV-1 regulatory protein Rev is another example for a cofactor required for mRNA export. In its absence, unspliced or incompletely spliced viral mRNAs coding for the proteins Gag, Pol and Env are not transported into the cytoplasm and thus viral replication fails, making Rev-mediated RNA export in HIV infection an interesting process to interfere with by drug treatment [61,62].

Besides the established role in nucleocytoplasmic trafficking, further investigations clarified the role of CRM1 in different cellular processes. Additional functions include opposing the effects of Impβ in mitosis [63] and a role in mitotic progression as it localizes to kinetochores and binds to RanGAP1 and RanBP2 in a RanGTP-dependent manner. Moreover, CRM1 has additional effects on the definition of kinetochore fibers and in chromosome segregation during mitosis. In particular, CRM1 activity in metaphase and later anaphase changes repartitioning of RanGTP and consequently also of effectors on kinetochores and centrosomes [63,64,65,66,67,68,69].

### 3.2. Conformational States of CRM1 during Nucleocytoplasmic Transport

Structural investigations of CRM1 in different assembly states enabled insight into the local structural rearrangements of CRM1 that stabilize overall conformational changes of CRM1 between the individual steps of a nucleocytoplasmic transport cycle.

**Figure 3:**
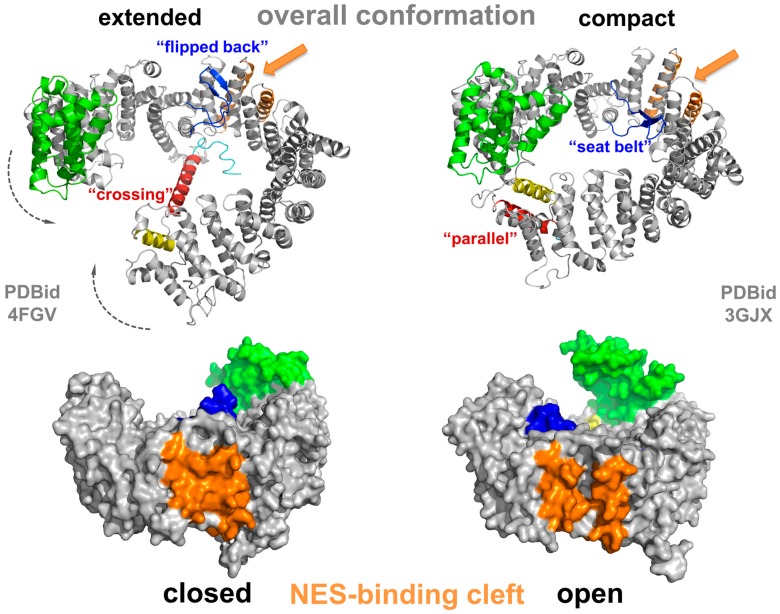
Structural changes of CRM1 between the extended and compact conformation. Structural overview illustrating the conformational changes of CRM1 during an export cycle. The conformations depicted here thus highlight the changes in two conformational states of CRM1 (**grey**) with respect to the overall shape (extended *versus* compact) as well as the positional changes of the CRIME-domain (**green**), the acidic loop (**blue**), the *C*-terminal helix (HEAT helix 21B, **red**) and the conformation of the NES-binding cleft (**orange**) during the transport cycle. See main text for details.

CRM1 consists of 21 HEAT repeats, in such an arrangement that the A helices form the convex outer surface of the protein, and the B helices form the concave inner surface [70,71,72]. Their slightly tilted, consecutive arrangement results in an overall superhelical twist with a flexible pitch [72,73,74]. Structural investigations of CRM1 in the free state (e.g., cargo- and Ran-unbound form) have shown that it adopts various conformations at equilibrium [75,76]. Multiple conformations of the extended (free) form have recently been observed in crystal structures at reasonable resolution [75,77], revealing a superhelical conformation with no interaction of the *N*- and *C*-terminal regions (Figure 3, left panel). Interestingly, the last HEAT repeat, 21, is unusual, as it arranges in two different states. In the extended, cargo-free form of CRM1, helix 21B spans the molecule reaching the opposing side of the superhelix (Figure 3, left panel).

Here, it touches the base of HEAT 9 helices A and B [70,75], and thus CRM1 is incapable of RanGTP binding, which attaches within the superhelix (see below). Further investigations showed that a negatively charged stretch within the *C*-terminal residues following HEAT 21B forms electrostatic interactions with a basic patch on HEAT 12B in the vicinity of the acidic loop binding site and contributes to modulation of NES-binding cleft properties [78]. The acidic loop itself is a stretch of 26 residues and located between HEAT helices 9A and B. In the extended forms of CRM1, it is oriented in a “flipped back” conformation closely binding to the B-helices of CRM1 in the NES-binding region formed by HEATs 11 and 12 (Figure 3) [70,71,76,79,80].

The highest sequence identity between CRM1 and other members of the Impβ superfamily has been detected in the first three HEAT repeats, the CRIME domain (Figure 3), indicating its functional importance [48,49,72,79,81]. This region is required for the binding of RanGTP. In the nuclear compartment, binding of RanGTP to the CRIME-domain could trigger progressive encircling of RanGTP by CRM1. The binding is accompanied by a reduction of the helical pitch of CRM1 that leads to a displacement of helix 21B from its “crossing” orientation to a “parallel” orientation at the outside of CRM1 [71,82]. This in turn, results in a closed CRM1 structure and tight interaction of *N*- and *C*-terminal regions (Figure 3, right panel). The acidic loop is released from its “flipped back” conformation and arranges like a “seatbelt” with the tip of the loop contacting residues of HEATs on the opposing side of CRM1, thereby locking RanGTP on the N-terminal part of CRM1 (Figure 3) [71]. Consequently, mechanical strain on the NES-binding cleft decreases, leading to opening of the cleft and increased accessibility for NES-cargos. As a result, a stable export complex assembles, which may then traverse the NPC (Figure 2).

In the cytoplasm, this ternary complex encounters RanBPs, which increase RanGAP binding and the GTP-hydrolysis rate of Ran. One of them, namely RanBP1, is soluble, whereas RanBP2 (Nup358), is localized directly at the pore bound to the filaments emanating from the NPC. Modification of RanGAP by the Small Ubiquitin-like Modifier (SUMO) tethers it to RanBP2 [42,83]. Structural analysis revealed that binding of RanBPs to the CRM1-RanGTP-cargo complex induces significant changes in CRM1 [82]. The binding of RanBP1 fixes the *C*-terminal acidic DEDDDL sequence of Ran in a position leading to displacement of the acidic loop from CRM1 and in turn interacts with the Ran switch I loop and the adjacent CRM1 surface [82]. The relocalization of the acidic loop in the proximity of HEAT helices 11B and 12B on the inside of the CRM1 ring is thought to induce structural changes at CRM1 HEATs 11 and 12, leading to a constriction of the NES-binding cleft and thus release of the NES-cargo [82].

The RanGTP-RanBP1 subcomplex has to dissociate from the export complex in order to interact with RanGAP. Subsequently, GTP is hydrolyzed to GDP by Ran aided by RanGAP and RanBPs with the resulting RanGDP exhibiting a lower affinity towards CRM1. CRM1 lacking any binding partners translocates back into the nucleus for another round of export.

### 3.3. NES Recognition by CRM1 and Export of (Proto-) Oncoproteins or Tumor Suppressors

**Figure 4:**
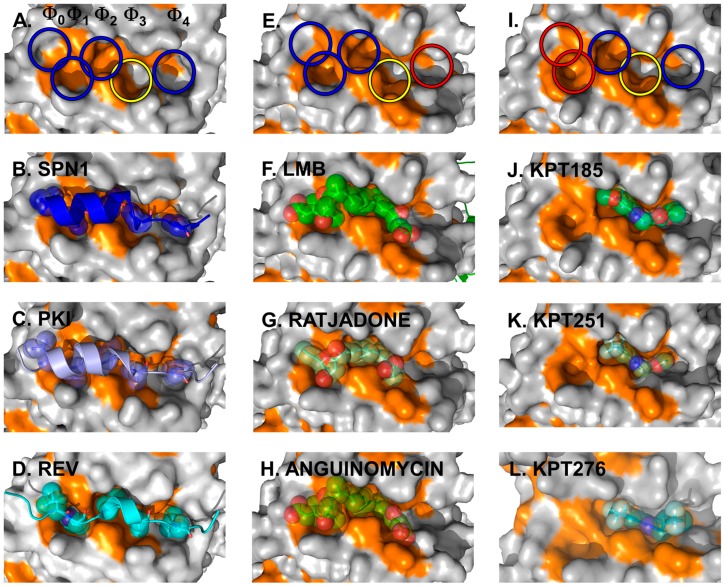
Cargo and inhibitor binding via the NES-binding cleft. Structural features of the NES-binding cleft of CRM1 is shown at the top. The five hydrophobic binding pockets for the interacting residues of the NESes are indicated by circles, with blue and yellow indicating the occupied sites and red circles highlighting the sites unused by the inhibitors depicted in the panels below. Additionally, the yellow circle indicates the position of Cys528 in human CRM1 and the S539C mutation in yeast CRM1 used for covalent binding of the inhibitors. Left panels: three NESes differing with respect to the spacing in between the residues determining binding within a rigid NES-binding pocket are shown. Detailed view of the NES-binding clefts of CRM1 bound to the SPN1-NES (**A**,**B**) (PDBid: 3GJX), PKI-NES; (**C**) (PDBid: 3NBY); and Rev-NES (**D**) (PDBid: 3NBZ). The SPN1-NES has been removed in (**A**) for clarity reasons to show the dimensions of the cleft and the respective Φ-pockets. Note that the key residues of all three NESes occupy identical Φ-pockets and differences in the Φ-spacing are compensated by a different arrangement of the NES-peptide main chain. Natural inhibitors of cargo binding bound to yeast CRM1 are shown in the middle panels. Blocking of the NES-binding cleft by CRM1-specific natural inhibitors: LMB (**E**,**F**) (PDBid: 4HAT); as well as Ratjadone (**G**) (PDBid: 4HAU); and Anguinomycin (**H**) (PDBid: 4HAV). LMB has been removed in (**E**) to show the dimensions of the occupied NES-binding cleft. Small synthetic inhibitors and their orientation in the NES-binding cleft are depicted on the right. KPT185 (**I**,**J**) (PDBid: 4GMX), KPT251; (**K**) (PDBid: 4GPT) and KPT276 (PDBid: 4WVF). KPT185 has been removed in (**I**) to show the dimensions and highlight the individual pockets of the occupied NES-binding cleft.

The crystal structures of full-length CRM1 in complex with RanGTP and/or the cargo Snurportin1 (SPN1) revealed for the first time how CRM1 and the NES of cargo interact on a structural level (Figure 4A,B) [70,71].

The CRM1 cargo SPN1 facilitates the import of UsnRNP core particles by bridging the interaction between the UsnRNPs and the actual import receptor Impβ. In this pathway, SPN1 specifically binds the modified 5ʹ-cap of the UsnRNP core particle *via* its cap-binding domain (CBD) [84]. For relocalization into the cytoplasm, SPN1 bears an N-terminally localized CRM1-dependent NES, which forms an amphipathic α-helix [71,80,85]. Within that α-helix, five hydrophobic key residues dock into corresponding hydrophobic pockets (named Φ0–Φ4) of the NES-binding cleft of CRM1 (Figure 4, left panels) [70,71]. In fact, the hydrophobic character, the size and the position of these Φ residues are important and essential for high-affinity binding of NES to CRM1. This is underlined by the observation that a single mutation of any of the Φ residues to a polar amino acid leads to a significantly weaker binding of a given NES [80]. Most strikingly, removal of the first methionine of the SPN1-NES occupying the Φ0 position entirely abolishes binding to CRM1, thereby reflecting its importance [71]. Cys528 (in human CRM1), which is known to be modified by Leptomycin B (LMB) and many other CRM1-blocking compounds, is located in the vicinity of the Φ3 position and thus in the central region of the CRM1 NES-binding cleft.

Subsequent structural analyses of additional NESes, fused to the CBD of SPN1 (SPN1-chimeras) revealed that the identical pockets Φ0–Φ4 within a rigid NES-binding cleft of CRM1 are used for binding of the PKI and Rev NES [80]. As the five hydrophobic key residues of the three different NESes exhibit different spacings on the amino acid sequence level, this requires a different arrangement of the NES-peptide chain to compensate for such differences (Figure 4, left panels).

Databases like “NESdb” and “ValidNESs” are available, which archive NES-containing CRM1 cargoes. At t last count (July 2015), there were 265/262 entries in these databases referring to macromolecules that bind to CRM1 and hence are exported [57,58]. Since all of them are assumed to bind in the same hydrophobic Φ pockets, they most likely apply a similar binding mode.

In recent years, CRM1 has been identified as an export receptor for various (proto-) oncoproteins and tumor suppressor genes like p53 [9], BRCA1 [86], p21^CIP^ [87], cyclin D1 [88], APC [17,89,90], Bok [91], forkhead box (FoxO) proteins [92,93,94], nucleophosmin [66,95,96], N-WASP [97], as well as the established drug target topoisomerase I/II [98,99,100]. The observed differences between normal and malignant cells with respect to the localization of proteins that function as oncoproteins makes CRM1 itself an interesting target in molecular oncology and therapeutics (Figure 1). Such changes in the localization of (proto-) oncoproteins and their deregulation may be caused either by an increase of cellular CRM1 levels that influence their distribution pattern by competition or due to any interference with the protein-CRM1 interaction. There are several possibilities for the latter case, like mutations (e.g., nucleophosmin, CRM1 [101]), phosphorylation (e.g., p27^KIP^, Rb, p53 [13,102,103,104,105]), ubiquitination and sumoylation (e.g., p53 [106,107,108]) or NES unmasking (e.g., INI1, NF-AT, p53, BRCA2/RAD51 [9,109,110,111]). However, as a consequence, both possibilities may finally lead directly or indirectly to a deregulation of nuclear export of tumor suppressor proteins or (proto-) oncoproteins (see Figure 1 and below).

A well-known drug target (e.g., targeted by doxorubicin and etoposide) against multiple myeloma is Topoisomerase IIα (Topo IIα), a nuclear protein, which is essential for DNA replication, transcription, chromatid separation as well as chromatin condensation [112,113,114]. For nucleocytoplasmic shuttling, Topo IIα contains both an NLS in its *C*-terminal domain, as well as two NES sequences in the central catalytic domain [99]. It has been shown that at increased cell densities and in myeloma cells, Topo IIα is exported to the cytoplasm in a CRM1-dependent fashion rendering the cells resistant to Topo IIα-specific inhibitors, which rather act on the DNA-bound nuclear protein [99,115,116]. Using a combination of Topo IIα inhibitors and efficient, non-acute toxic CRM1 inhibitors allows keeping the protein in the nucleus and hence sensitize it for Topo IIα inhibitor treatment [117,118].

The breast cancer-associated protein, BARD1 (BRCA1-associated RING domain protein), co-localizes with BRCA1 in nuclear foci [119]. After DNA damage, the two proteins form a stable heterodimer implicated in multiple nuclear functions like DNA repair, protein ubiquitination and control of mRNA processing [120,121,122]. Additionally, it has been observed that BRCA1 mislocalizes to the cytoplasm in cancer cells but not in normal cells. Later it was shown that BARD1 has BRCA1-independent pro-apoptotic activity in the cytoplasm. Both, BRCA1 as well as BARD1 harbor NESes [123,124], which are part of the BRCA1/BARD1 dimerization surface and thus are masked when both proteins bind to each other. Disruption of this interaction leads to cytoplasmic accumulation and increased apoptosis. However, it seems that in such cells, nuclear import of BRCA1/BARD1 is impaired rather than nuclear export of BARD1, thereby explaining its cytoplasmic accumulation and thus cancer development [121,123,125].

The inhibitor of apoptosis Survivin is highly abundant in human tumors and in fetal cells but absent in normal cells. It has been shown that Survivin contains a leucine-rich NES (amino acids 89–98) for CRM1 export [126,127] but lacks a classical import signal (NLS). Survivin has different functions in cell viability and cell division. The cytoplasmic (e.g., exported) form controls cell viability as it inhibits caspase activation and thus prevents apoptosis. This effect is thought to contribute significantly to the fast growth and apoptotic resistance of tumor cells. In contrast, nuclear Survivin regulates cell division as it is part of the chromosomal passenger complex, which coordinates essential chromosomal and cytoskeletal events during mitosis [126,127,128,129,130].

The cyclin-dependent kinase inhibitor p27^KIP^ is an important regulator of the cell cycle. During cell cycle progression, it binds and inhibits cyclin/cyclin-dependent kinase (CDK) complexes in the nucleus and thus stops or slows down cell division at the G1 stage [131,132,133]. For example, interaction of p27^KIP^ with cyclin D and CDK4 inhibits the kinase activity and thus prevents phosphorylation and inactivation of the transcriptional repressor, retinoblastoma tumor suppressor protein (Rb) [134,135]. Notably, the activity of p27^KIP^ itself is subjected to regulation on multiple levels like transcription, translation, proteolysis and nuclear export [104]. For CRM1-dependent export, phosphorylation of p27^KIP^ Ser10 by other kinases plays an essential role [136]. As cytoplasmic p27^KIP^ is no longer able to inhibit cyclin/CDK complexes, Rb is consequently phosphorylated and inactivated, resulting in expression of multiple factors and promotion of fast cell cycle progression [137]. This, in turn, is highly correlated with a high tumor grade, poor prognosis and increased metastasis in different subsets of carcinomas like breast-, cervix, esophagus and uterus carcinomas as well as in lymphomas and leukemia [138,139,140].

Apart from a direct interference of CRM1 (proto-) oncogene interaction by the mentioned effects, elevated CRM1 expression levels in tumor cells can cause nuclear export to be deregulated. Indeed, CRM1 protein expression level was shown to be a prognostic indicator for various cancers and is also correlated with increased metastasis, histological grade, increased tumor size, and decreased progression-free and overall survival. In particular, elevated CRM1 expression correlates with poor clinical outcome in ovarian- [141], pancreatic- [142,143], kidney- [144] and cervical cancers [145], as well as gastric carcinomas [146], osteosarcoma [147], glioma [148] as well as leukemia [149,150]. In addition, mantle cell lymphoma [151], multiple myeloma [152,153] and melanoma [154] have been shown to be accompanied by elevated CRM1 levels [118,143,150,155,156].

Although it is a more global strategy to interfere with CRM1-dependent transport processes as it affects all proteins bearing a canonical NES in all cells, it seems to be a promising idea to use natural or synthetic compounds to block CRM1 and thus interfere with transport and localization of cancer-related proteins. Interestingly, the effect of CRM1 inactivation seems to have a more pronounced effect on cancer cells leading to increased apoptosis than on normal cells that tolerate such compounds to a certain degree.

Not only CRM1 but also other transport factors exhibit altered expression and functions in cancer cells. A role as prognostic biomarker has been determined for Exportin 7 in ovarian cancers [157]. Recently, it has been shown that Impβ expression is increased in several malignant tumors such as cervical tumors and malignant peripheral nerve sheath tumors (MPNSTs) as well as in breast, gastritic, neck, lung and ovarian cancers [145,158,159,160,161,162]. Impβ up-regulation has been shown to promote cell proliferation in gastritic and cervical cancer cells [145,158]. Also, one of the adaptor molecules Karyopherin α2 bridging the interaction between Impβ and NLS-bearing cargo is a potential biomarker in multiple cancers reviewed in [163]. A role as prognostic biomarker has been determined for the small GTPase Ran in ovarian and colorectal cancer [157,164]. Ran promotes cancer cell metastasis by interaction with Txl-2 [165]. Moreover, it has been shown to promote proliferation of pancreatic cancer cells [166] and is a potential therapeutic target in diffuse large B-Cell lymphoma and other cancer cells with specific defects [167,168].

In line with these observations, progress with respect to altering transport processes by drugs has been made. For example, treatment of MPNST cells with an inhibitor of S-adenosyl-methionine-dependent methyl-transferases, 3-deazaneplanocin A (DZNep) impaired cell viability and proliferation and reduced Impβ protein levels [159]. The Impβ1-specific inhibitor Importazole inhibits Impβ1’s role in nuclear import [169] and decreases the viability of malignant breast tumor cells much more than that of the non-transformed counterpart [162].

### 3.4. Drug Binding to CRM1

The first compound identified as the most potent CRM1 inhibitor is the drug Leptomycin B (LMB) [49,50,61,170], which is able to block Rev function and HIV-1 replication [61,171].

All the natural compounds identified thus far have an α,β-unsaturated δ-lactone ring in common. The *Streptomyces spp*. natural products LMB and Kazusamycin were originally characterized as antifungal and antitumor agents [61,172,173,174,175]. An additional member of the family, Anguinomycin, was isolated as a natural product from *Streptomyces spec*. and later on also produced by total chemical synthesis [176,177,178,179]. Interestingly, a simple α,β-unsaturated lactone analog with a truncated polyketide chain was shown to retain most of the biological activity [178].

Ratjadone belongs to another group of inhibitors and was isolated from the myxobacterium *Sorangium cellulosum* strain So ce360 [180,181]. Chemical total synthesis was established, providing the tools for the production of variants [182,183]. Ratjadone blocks export by employing the same mechanism as LMB [184,185], hence crystal structure analyses revealed that it uses the identical binding pockets of the CRM1 NES-binding domain. In addition, it has recently been shown to block the Rev/CRM1 export pathway [186].

**Figure 5:**
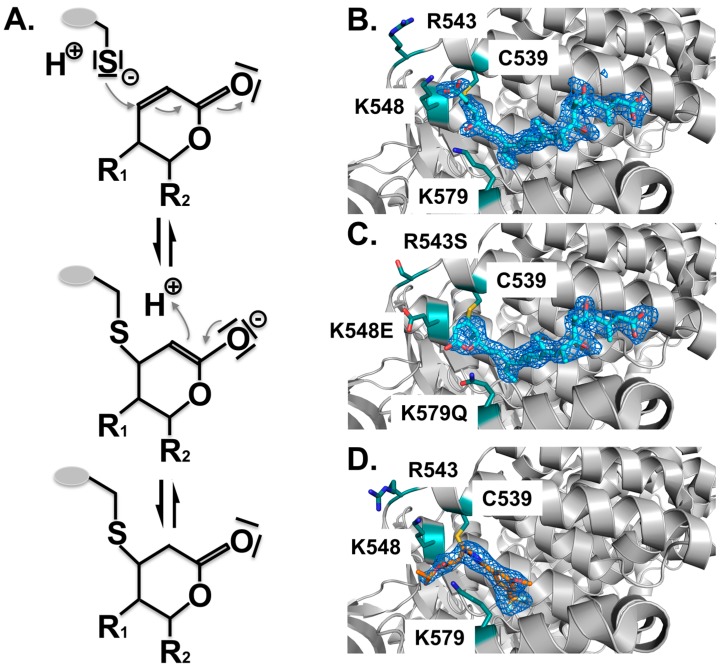
Covalent binding of inhibitors to the NES-binding cleft of CRM1. All inhibitors characterized thus far are covalently attached to the reactive cysteine C539 by a Michael-type addition. (**A**) Mechanism of the Michael addition of the cysteine to the lactone ring of CRM1 inhibitors. Subsequently, in the α,β-unsaturated δ-lactone ring containing compounds, a nucleophilic attack of a water molecule leads to lactone hydrolysis in a subsequent step due to the neighboring basic residues that form an oxyanion hole stabilizing the transition state of the reaction (not depicted). For compounds containing an α,β-unsaturated δ-lactone ring, binding is irreversible due to the CRM1-catalyzed opening of the lactone ring. Structural arrangement of the NES-binding cleft of CRM1 shown in cartoon mode (**grey**); (**B**) Binding of LMB (cyan) to CRM1 results in hydrolysis of the lactone ring due to neighboring basic residues indicated (teal). The mFo-DFc omit map contoured at a level of 3.0 sigma, clearly shows the arrangement of the hydrolyzed LMB; (**C**) Mutation of the three basic residues prevents ring opening of LMB (cyan); (**D**) By contrast, binding of the novel compounds lacking the lactone ring, is slowly reversible. KPT185 interacts with Cys528, but no other changes are observed.

Structure analysis revealed the mode of binding of LMB and the related inhibitors Anguinomycin A and Ratjadone A to the NES-binding pocket (Figure 4E–H). Moreover, deeper analysis revealed that all three show an unexpected mechanism of inhibition, involving covalent conjugation of the α,β-unsaturated δ-lactone ring to Cys539 of CRM1 [172,185]. The subsequent CRM1-catalyzed hydrolysis of the natural products’ lactone ring, which is mediated by basic residues (Lys or Arg) positioned near the reactive cysteine, renders the binding irreversible (Figure 5) [187]. All three natural compounds occupy the same space in the NES-binding cleft of CRM1, namely four of the five hydrophobic pockets used by the SPN1-NES (Φ0 to Φ3) for binding, leaving Φ4 vacant (Figure 4, middle panels, red circle). The reactive cysteine (aa 528 in human and aa S539C mutation in yeast) lies in the vicinity of the hydrophobic pocket Φ3, and binding of LMB restricts binding of NESes by spatial competition (Figure 3) [172,173,187]. Interestingly, covalent conjugation is not strictly required for LMB binding to the CRM1 groove, because the groove is also open in a complex of LMB with CRM1 that lacks the reactive cysteine, but seems highly flexible, as indicated by weak electron density [187].

For LMB, murine xenograft cancer models revealed a modest efficacy (CI-940) [188] and clinical phase I trial confirmed the results but also pinpointed severe toxicities, including anorexia and malaise [189]. Moreover, the identification of the specific inhibition of CRM1 export function by the drug LMB [49,50,61,170] and recent structural understanding of the mode of cargo recognition within the NES-binding cleft and the inhibition of exactly that binding cleft by LMB [70,71,187], led to the development of novel therapeutics [149,156,190]).

Semisynthetic products of LMB coined Nuclear Export Inhibitors (NEIs) revealed improved therapeutic capabilities by maintaining the high potency observed for LMB, as they are better tolerated *in vivo*, and show significant efficacy in multiple mouse xenograft models. These NEIs are thought to have potential as CRM1 inhibitors and potent anticancer agents [190].

Another synthetic small-molecule and CRM1 inhibitor, an analog of a class of compounds called N-azolylacrylates, was developed in a study by Daelemans *et al.* [191]. It exhibits the same cellular effects, namely prevents nuclear export of the HIV-1 Rev protein and is a highly specific inhibitor of CRM1. Like LMB, this compound (PKF050-638) interferes with the NES-binding cleft cysteine and prevents binding of the nuclear export signal [191]. The further development led to Small Inhibitors of Nuclear Export (SINEs) that are similar to the N-azolylacrylate structures [192]. Three of those SINEs (KPT185, KPT251 and KPT276), which all share a trifluoromethyl phenyl triazole scaffold, have been crystallized in a complex with CRM1 [149,156,193]. Crystal structure analysis revealed that they occupy only three of the five hydrophobic pockets (Φ2–Φ4) centered on the reactive cysteine leaving Φ0 and Φ1 vacant (Figure 4J–L). The structural comparison of the interaction network of the natural compounds like LMB, Anguinomycin A and Ratjadone A with CRM1 on the one side [187] and synthetic compounds e.g., KPT185 and KPT251 on the other, revealed an additional important property of the synthetic compounds. In the synthetic compounds, the Michael acceptors (an isopropyl acrylate in KPT185 and an alkyl-oxadiazole in KPT251) are not hydrolyzed when bound to wild-type CRM1, thus they may bind in a slowly reversible fashion into the NES-binding cleft [187]. This reversibility of binding could contribute to the reduced side effects observed in *in vivo* studies, which is in contrast to the irreversible binding of the natural compounds. The putative weak interactions of NESes to the vacant sites Φ0 and Φ1 of the NES-binding cleft could increase the rate of synthetic inhibitor release, enabling cargo binding to an extent that is sufficient for normal, but not for malignant, cell survival.

These small molecule inhibitors or derivatives thereof are being used in clinical trials in patients with both hematological malignancies [139,149] and solid tumors [153]. Antitumor effects of KPT185 and its clinical equivalent KPT276 have been shown in cancer cells and xenografts [194]. An improved version is KPT330, an oral drug currently undergoing phase I studies in patients with advanced, relapsed, and refractory solid tumors, hematological malignancies, and sarcoma [139,153,195]. Preclinical evaluation of bioavailable SINE KPT335 has been performed in canine cancer cells and dogs [196].

There is a large number of additional nuclear export inhibitors from both natural and synthetic sources available. Natural compounds include Prostaglandins [197], the spice curcumin from the plant *Curcuma longa*, which is already in clinical trials [198,199,200], or compounds from the plants *Valerianae sp.* [171,201,202]. In addition, Plumbagin, a bicyclic naphtoquinone [203], and Piperlongumine, a natural alkaloid of the long pepper [204], have been shown to inhibit CRM1-mediated nuclear export. Moreover, the cytotoxic styryl-lactone Goniothalamin from the family of *Annonaceae* [205,206,207,208,209], 19S-19-Acetoxychavicol acetate isolated from *Alpinia galangal* [210,211], Callystatin A from the marine sponge *Callyspongia truncata* [212,213,214] and Leptofuranins from *Streptomyces tanashiensi*s [215,216] have been described. Compounds resulting from screening experiments are CBS9106, a novel reversible oral CRM1 inhibitor with CRM1-degrading activity [217], as well as multiple compounds that have been identified in screens examining nuclear export of FOXO proteins or inhibit the activation-dependent nuclear export of the p38 kinase substrate MAPK-activated protein kinase 2 (MK2) [218,219].

As these compounds block CRM1-dependent export and influence the cellular distribution pattern of the proteins/oncoproteins/tumor suppressor proteins, they modulate the fate of cancer cells by decreasing their survival rate [139,143,144,149,151,154,155,220]. To this end, treatment with KPT330 has been shown to result in the nuclear enrichment of various proteins e.g., p53, and p21, which then are able to perform their cellular function decreasing the survival rate of the cancer cells as shown in diffuse malignant peritoneal mesothelioma, renal cell carcinoma and leukemias [195,221,222,223]. Recently, a novel drug (S109, a derivative of CBS9106) was shown to inhibit proliferation and arrest colorectal cancer cells by nuclear retention of tumor suppressor proteins like p21, p27 and FOXO, by reversibly binding to CRM1 and to decrease the CRM1 level using the proteasomal pathway [224]. For detailed information on the compounds and their effects on various cancers, we refer to excellent recent reviews [225,226,227,228].

As an additional effect, the CRM1 inhibitors may sensitize resistant cancer cells for other drugs, e.g., as shown by Topoisomerase in multiple myeloma, which has to be localized in the nucleus in order to be sensitive for doxorubicin and etoposide treatment [117]. Along this line, CRM1 inhibition by KPT330 enhances the antitumor activity of Gemcitabine in pancreatic cancer [229] or of Ibrutinib in chronic lymphocytic leukemia [230]. Overall, this supports the observation that blocking CRM1 sensitizes cancer cells to other drugs by preventing export of additional tumor suppressors or cell cycle inhibitors.

## 4. Conclusions, Outlook, Pending Issues

The idea of using CRM1 as a drug target to battle cancer is based on the observation that treatment of cancer cells with natural drugs leads to a prolonged block of nuclear export and subsequent apoptosis of cancer cells. In contrast, in normal cells, these drugs, although they induced cell cycle arrest, do not lead to apoptosis. Recent investigations identified novel synthetic and semi-synthetic compounds with reduced side effects.

Understanding the molecular differences of CRM1-dependence (and influence of inhibitors) in healthy and cancerogenic cells will help to design compounds that are more specific.

In the future, improved understanding of the NPC-CRM1 interaction and the transition process through the NPC itself might also be used for selective inhibition of nucleocytoplasmic transport. This could be achieved by specifically targeting the CRM1-Nup interactions, e.g., Nup214, Nup98 or hCG1 or by modulation of export of only a subset of NES-cargoes [231,232,233,234,235,236,237,238,239].

## References

[1] Dobbelstein M., Moll U. (2014). Targeting tumour-supportive cellular machineries in anticancer drug development. Nat. Rev. Drug Discov..

[2] Hanahan D., Weinberg R.A. (2000). The hallmarks of cancer. Cell.

[3] Hanahan D., Weinberg R.A. (2011). Hallmarks of cancer: The next generation. Cell.

[4] Goldstein I., Madar S., Rotter V. (2012). Cancer research, a field on the verge of a paradigm shift?. Trends Mol. Med..

[5] Weinberg R.A. (2014). Coming full circle-from endless complexity to simplicity and back again. Cell.

[6] Faustino R.S., Nelson T.J., Terzic A., Perez-Terzic C. (2007). Nuclear transport: Target for therapy. Clin. Pharmacol. Ther..

[7] Turner J.G., Dawson J., Sullivan D.M. (2012). Nuclear export of proteins and drug resistance in cancer. Biochem. Pharmacol..

[8] Hill R., Cautain B., de Pedro N., Link W. (2014). Targeting nucleocytoplasmic transport in cancer therapy. Oncotarget.

[9] Stommel J.M., Marchenko N.D., Jimenez G.S., Moll U.M., Hope T.J., Wahl G.M. (1999). A leucine-rich nuclear export signal in the p53 tetramerization domain: Regulation of subcellular localization and p53 activity by nes masking. EMBO J..

[10] Foo R.S., Nam Y.J., Ostreicher M.J., Metzl M.D., Whelan R.S., Peng C.F., Ashton A.W., Fu W., Mani K., Chin S.F. (2007). Regulation of p53 tetramerization and nuclear export by arc. Proc. Natl. Acad. Sci. USA.

[11] Muller P.A., Vousden K.H. (2014). Mutant p53 in cancer: New functions and therapeutic opportunities. Cancer Cell.

[12] Vousden K.H., Prives C. (2005). P53 and prognosis: New insights and further complexity. Cell.

[13] Jiao W., Datta J., Lin H.M., Dundr M., Rane S.G. (2006). Nucleocytoplasmic shuttling of the retinoblastoma tumor suppressor protein via cdk phosphorylation-dependent nuclear export. J. Biol. Chem..

[14] Kowalik A., Kopczynski J., Wypiorkiewicz E., Gozdz S., Mezyk R., Siedlecki J.A. (2013). Active transport of rb protein from the nucleus to the cytoplasm as one of the development mechanisms of her2-positive breast cancer. Pol. J. Pathol. Off. J. Pol. Soc. Pathol..

[15] Mittnacht S., Lees J.A., Desai D., Harlow E., Morgan D.O., Weinberg R.A. (1994). Distinct sub-populations of the retinoblastoma protein show a distinct pattern of phosphorylation. EMBO J..

[16] Stokke T., Erikstein B.K., Smedshammer L., Boye E., Steen H.B. (1993). The retinoblastoma gene product is bound in the nucleus in early g1 phase. Exp. Cell Res..

[17] Neufeld K.L., Nix D.A., Bogerd H., Kang Y., Beckerle M.C., Cullen B.R., White R.L. (2000). Adenomatous polyposis coli protein contains two nuclear export signals and shuttles between the nucleus and cytoplasm. Proc. Natl. Acad. Sci. USA.

[18] Neufeld K.L., White R.L. (1997). Nuclear and cytoplasmic localizations of the adenomatous polyposis coli protein. Proc. Natl. Acad. Sci. USA.

[19] Powell S.M., Zilz N., Beazer-Barclay Y., Bryan T.M., Hamilton S.R., Thibodeau S.N., Vogelstein B., Kinzler K.W. (1992). APC mutations occur early during colorectal tumorigenesis. Nature.

[20] Henderson B.R., Fagotto F. (2002). The ins and outs of APC and β-catenin nuclear transport. EMBO Rep..

[21] Jamieson C., Sharma M., Henderson B.R. (2014). Targeting the beta-catenin nuclear transport pathway in cancer. Semin. Cancer Biol..

[22] Crabtree G.R., Olson E.N. (2002). Nfat signaling: Choreographing the social lives of cells. Cell.

[23] Mancini M., Toker A. (2009). Nfat proteins: Emerging roles in cancer progression. Nat. Rev. Cancer.

[24] Muller M.R., Rao A. (2010). Nfat, immunity and cancer: A transcription factor comes of age. Nat. Rev. Immunol..

[25] Pan M.G., Xiong Y., Chen F. (2013). Nfat gene family in inflammation and cancer. Curr. Mol. Med..

[26] Qin J.J., Nag S., Wang W., Zhou J., Zhang W.D., Wang H., Zhang R. (2014). Nfat as cancer target: Mission possible?. Biochim. Biophys. Acta.

[27] Lugo T.G., Pendergast A.M., Muller A.J., Witte O.N. (1990). Tyrosine kinase activity and transformation potency of Bcr-Abl oncogene products. Science.

[28] Cilloni D., Saglio G. (2012). Molecular pathways: Bcr-Abl. Clin. Cancer Res..

[29] Drake J.M., Lee J.K., Witte O.N. (2014). Clinical targeting of mutated and wild-type protein tyrosine kinases in cancer. Mol. Cell Biol..

[30] Brohawn S.G., Partridge J.R., Whittle J.R., Schwartz T.U. (2009). The nuclear pore complex has entered the atomic age. Structure.

[31] Hurt E., Beck M. (2015). Towards understanding nuclear pore complex architecture and dynamics in the age of integrative structural analysis. Curr. Opin. Cell Biol..

[32] Kabachinski G., Schwartz T.U. (2015). The nuclear pore complex—Structure and function at a glance. J. Cell Sci..

[33] Andrade M.A., Bork P. (1995). Heat repeats in the huntington’s disease protein. Nat. Genet..

[34] Andrade M.A., Perez-Iratxeta C., Ponting C.P. (2001). Protein repeats: Structures, functions, and evolution. J. Struct. Biol..

[35] Izaurralde E., Kutay U., von Kobbe C., Mattaj I.W., Gorlich D. (1997). The asymmetric distribution of the constituents of the ran system is essential for transport into and out of the nucleus. EMBO J..

[36] Ohtsubo M., Okazaki H., Nishimoto T. (1989). The rcc1 protein, a regulator for the onset of chromosome condensation locates in the nucleus and binds to DNA. J. Cell Biol..

[37] Bischoff F.R., Maier G., Tilz G., Ponstingl H. (1990). A 47-kda human nuclear protein recognized by antikinetochore autoimmune sera is homologous with the protein encoded by rcc1, a gene implicated in onset of chromosome condensation. Proc. Natl. Acad. Sci. USA.

[38] Uchida S., Sekiguchi T., Nishitani H., Miyauchi K., Ohtsubo M., Nishimoto T. (1990). Premature chromosome condensation is induced by a point mutation in the hamster rcc1 gene. Mol. Cell Biol..

[39] Smith A.E., Slepchenko B.M., Schaff J.C., Loew L.M., Macara I.G. (2002). Systems analysis of ran transport. Science.

[40] Kalab P., Weis K., Heald R. (2002). Visualization of a ran-gtp gradient in interphase and mitotic xenopus egg extracts. Science.

[41] Bischoff F.R., Klebe C., Kretschmer J., Wittinghofer A., Ponstingl H. (1994). RanGAP1 induces GTPase activity of nuclear ras-related ran. Proc. Natl. Acad. Sci. USA.

[42] Mahajan R., Delphin C., Guan T., Gerace L., Melchior F. (1997). A small ubiquitin-related polypeptide involved in targeting RanGAP1 to nuclear pore complex protein RanBP2. Cell.

[43] Matunis M.J., Coutavas E., Blobel G. (1996). A novel ubiquitin-like modification modulates the partitioning of the Ran-GTPase-activating protein RanGAP1 between the cytosol and the nuclear pore complex. J. Cell Biol..

[44] Culjkovic-Kraljacic B., Baguet A., Volpon L., Amri A., Borden K.L. (2012). The oncogene eIF4e reprograms the nuclear pore complex to promote mrna export and oncogenic transformation. Cell Rep..

[45] Culjkovic-Kraljacic B., Borden K.L. (2013). Aiding and abetting cancer: Mrna export and the nuclear pore. Trends Cell Biol..

[46] Adachi Y., Yanagida M. (1989). Higher order chromosome structure is affected by cold-sensitive mutations in a schizosaccharomyces pombe gene CRM1+ which encodes a 115-kd protein preferentially localized in the nucleus and its periphery. J. Cell Biol..

[47] Fornerod M., Boer J., van Baal S., Morreau H., Grosveld G. (1996). Interaction of cellular proteins with the leukemia specific fusion proteins dek-can and set-can and their normal counterpart, the nucleoporin can. Oncogene.

[48] Fornerod M., van Deursen J., van Baal S., Reynolds A., Davis D., Murti K.G., Fransen J., Grosveld G. (1997). The human homologue of yeast CRM1 is in a dynamic subcomplex with can/Nup214 and a novel nuclear pore component Nup88. EMBO J..

[49] Fornerod M., Ohno M., Yoshida M., Mattaj I.W. (1997). CRM1 is an export receptor for leucine-rich nuclear export signals. Cell.

[50] Fukuda M., Asano S., Nakamura T., Adachi M., Yoshida M., Yanagida M., Nishida E. (1997). CRM1 is responsible for intracellular transport mediated by the nuclear export signal. Nature.

[51] Neville M., Stutz F., Lee L., Davis L.I., Rosbash M. (1997). The importin-beta family member CRM1p bridges the interaction between rev and the nuclear pore complex during nuclear export. Curr. Biol..

[52] Ossareh-Nazari B., Bachelerie F., Dargemont C. (1997). Evidence for a role of CRM1 in signal-mediated nuclear protein export. Science.

[53] Stade K., Ford C.S., Guthrie C., Weis K. (1997). Exportin 1 (CRM1p) is an essential nuclear export factor. Cell.

[54] Kehlenbach R.H., Dickmanns A., Gerace L. (1998). Nucleocytoplasmic shuttling factors including ran and CRM1 mediate nuclear export of nfat *in vitro*. J. Cell Biol..

[55] Fischer U., Huber J., Boelens W.C., Mattaj I.W., Luhrmann R. (1995). The HIV-1 rev activation domain is a nuclear export signal that accesses an export pathway used by specific cellular rnas. Cell.

[56] Wen W., Meinkoth J.L., Tsien R.Y., Taylor S.S. (1995). Identification of a signal for rapid export of proteins from the nucleus. Cell.

[57] Fu S.C., Huang H.C., Horton P., Juan H.F. (2013). Validness: A database of validated leucine-rich nuclear export signals. Nucleic Acids Res..

[58] Xu D., Farmer A., Collett G., Grishin N.V., Chook Y.M. (2012). Sequence and structural analyses of nuclear export signals in the nesdb database. Mol. Biol. Cell.

[59] Ohno M., Segref A., Bachi A., Wilm M., Mattaj I.W. (2000). Phax, a mediator of u snrna nuclear export whose activity is regulated by phosphorylation. Cell.

[60] Segref A., Mattaj I.W., Ohno M. (2001). The evolutionarily conserved region of the U snRNA export mediator phax is a novel rna-binding domain that is essential for u snrna export. RNA.

[61] Wolff B., Sanglier J.J., Wang Y. (1997). Leptomycin b is an inhibitor of nuclear export: Inhibition of nucleo-cytoplasmic translocation of the human immunodeficiency virus type 1 (HIV-1) rev protein and rev-dependent mrna. Chem. Biol..

[62] Booth D.S., Cheng Y., Frankel A.D. (2014). The export receptor CRM1 forms a dimer to promote nuclear export of HIV RNA. eLife.

[63] Roscioli E., di Francesco L., Bolognesi A., Giubettini M., Orlando S., Harel A., Schinina M.E., Lavia P. (2012). Importin-beta negatively regulates multiple aspects of mitosis including RanGAP1 recruitment to kinetochores. J. Cell Biol..

[64] Di Fiore B., Ciciarello M., Lavia P. (2004). Mitotic functions of the Ran-GTPase network: The importance of being in the right place at the right time. Cell Cycle.

[65] Arnaoutov A., Azuma Y., Ribbeck K., Joseph J., Boyarchuk Y., Karpova T., McNally J., Dasso M. (2005). CRM1 is a mitotic effector of ran-gtp in somatic cells. Nat. Cell Biol..

[66] Wang W., Budhu A., Forgues M., Wang X.W. (2005). Temporal and spatial control of nucleophosmin by the ran-CRM1 complex in centrosome duplication. Nat. Cell Biol..

[67] Torosantucci L., de Luca M., Guarguaglini G., Lavia P., Degrassi F. (2008). Localized rangtp accumulation promotes microtubule nucleation at kinetochores in somatic mammalian cells. Mol. Biol. Cell.

[68] Roscioli E., Bolognesi A., Guarguaglini G., Lavia P. (2010). Ran control of mitosis in human cells: Gradients and local signals. Biochem. Soc. Trans..

[69] Neuber A., Franke J., Wittstruck A., Schlenstedt G., Sommer T., Stade K. (2008). Nuclear export receptor XPO1/CRM1 is physically and functionally linked to the spindle pole body in budding yeast. Mol. Cell Biol..

[70] Dong X., Biswas A., Suel K.E., Jackson L.K., Martinez R., Gu H., Chook Y.M. (2009). Structural basis for leucine-rich nuclear export signal recognition by CRM1. Nature.

[71] Monecke T., Guttler T., Neumann P., Dickmanns A., Gorlich D., Ficner R. (2009). Crystal structure of the nuclear export receptor CRM1 in complex with snurportin1 and rangtp. Science.

[72] Monecke T., Dickmanns A., Ficner R. (2014). Allosteric control of the exportin CRM1 unraveled by crystal structure analysis. FEBS J..

[73] Zachariae U., Grubmuller H. (2006). A highly strained nuclear conformation of the exportin CSE1P revealed by molecular dynamics simulations. Structure.

[74] Zachariae U., Grubmuller H. (2008). Importin-beta: Structural and dynamic determinants of a molecular spring. Structure.

[75] Monecke T., Haselbach D., Voβ B., Russek A., Neumann A., Thomson E., Hurt E., Zachariae U., Stark H., Grubmüller H. (2013). Structural basis for cooperativity of CRM1 export complex formation. Proc. Natl. Acad. Sci. USA.

[76] Dolker N., Blanchet C.E., Voss B., Haselbach D., Kappel C., Monecke T., Svergun D.I., Stark H., Ficner R., Zachariae U. (2013). Structural determinants and mechanism of mammalian CRM1 allostery. Structure.

[77] Saito N., Matsuura Y. (2013). A 2.1-A-resolution crystal structure of unliganded CRM1 reveals the mechanism of autoinhibition. J. Mol. Biol..

[78] Fox A.M., Ciziene D., McLaughlin S.H., Stewart M. (2011). Electrostatic interactions involving the extreme C terminus of nuclear export factor CRM1 modulate its affinity for cargo. J. Biol. Chem..

[79] Petosa C., Schoehn G., Askjaer P., Bauer U., Moulin M., Steuerwald U., Soler-Lopez M., Baudin F., Mattaj I.W., Muller C.W. (2004). Architecture of CRM1/exportin1 suggests how cooperativity is achieved during formation of a nuclear export complex. Mol. Cell.

[80] Guttler T., Madl T., Neumann P., Deichsel D., Corsini L., Monecke T., Ficner R., Sattler M., Gorlich D. (2010). Nes consensus redefined by structures of pki-type and rev-type nuclear export signals bound to CRM1. Nat. Struct. Mol. Biol..

[81] Gorlich D., Dabrowski M., Bischoff F.R., Kutay U., Bork P., Hartmann E., Prehn S., Izaurralde E. (1997). A novel class of rangtp binding proteins. J. Cell Biol..

[82] Koyama M., Matsuura Y. (2010). An allosteric mechanism to displace nuclear export cargo from CRM1 and rangtp by ranbp1. EMBO J..

[83] Matunis M.J., Wu J., Blobel G. (1998). Sumo-1 modification and its role in targeting the Ran-GTPase-activating protein, RanGAP1, to the nuclear pore complex. J. Cell Biol..

[84] Strasser A., Dickmanns A., Luhrmann R., Ficner R. (2005). Structural basis for M3G-cap-mediated nuclear import of spliceosomal usnrnps by snurportin1. EMBO J..

[85] Paraskeva E., Izaurralde E., Bischoff F.R., Huber J., Kutay U., Hartmann E., Luhrmann R., Gorlich D. (1999). CRM1-mediated recycling of snurportin 1 to the cytoplasm. J. Cell Biol..

[86] Rodriguez J.A., Henderson B.R. (2000). Identification of a functional nuclear export sequence in BRCA1. J. Biol. Chem..

[87] Hwang C.Y., Kim I.Y., Kwon K.S. (2007). Cytoplasmic localization and ubiquitination of p21(cip1) by reactive oxygen species. Biochem. Biophys. Res. Commun..

[88] Benzeno S., Diehl J.A. (2004). *C*-terminal sequences direct cyclin d1-CRM1 binding. J. Biol. Chem..

[89] Henderson B.R. (2000). Nuclear-cytoplasmic shuttling of APC regulates β-catenin subcellular localization and turnover. Nat. Cell Biol..

[90] Rosin-Arbesfeld R., Townsley F., Bienz M. (2000). The APC tumour suppressor has a nuclear export function. Nature.

[91] Bartholomeusz G., Wu Y., Ali Seyed M., Xia W., Kwong K.Y., Hortobagyi G., Hung M.C. (2006). Nuclear translocation of the pro-apoptotic BCL-2 family member bok induces apoptosis. Mol. Carcinog..

[92] Brunet A., Kanai F., Stehn J., Xu J., Sarbassova D., Frangioni J.V., Dalal S.N., de Caprio J.A., Greenberg M.E., Yaffe M.B. (2002). 14-3-3 transits to the nucleus and participates in dynamic nucleocytoplasmic transport. J. Cell Biol..

[93] Latre de Late P., Pepin A., Assaf-Vandecasteele H., Espinasse C., Nicolas V., Asselin-Labat M.L., Bertoglio J., Pallardy M., Biola-Vidamment A. (2010). Glucocorticoid-induced leucine zipper (GILZ) promotes the nuclear exclusion of FOXO3 in a CRM1-dependent manner. J. Biol. Chem..

[94] Howell J.J., Stoffel M. (2009). Nuclear export-independent inhibition of FOXA2 by insulin. J. Biol. Chem..

[95] Falini B., Bolli N., Shan J., Martelli M.P., Liso A., Pucciarini A., Bigerna B., Pasqualucci L., Mannucci R., Rosati R. (2006). Both carboxy-terminus nes motif and mutated tryptophan(s) are crucial for aberrant nuclear export of nucleophosmin leukemic mutants in NPMC+ AML. Blood.

[96] Yu Y., Maggi L.B., Brady S.N., Apicelli A.J., Dai M.S., Lu H., Weber J.D. (2006). Nucleophosmin is essential for ribosomal protein l5 nuclear export. Mol. Cell Biol..

[97] Suetsugu S., Takenawa T. (2003). Translocation of n-wasp by nuclear localization and export signals into the nucleus modulates expression of HSP90. J. Biol. Chem..

[98] Mirski S.E., Bielawski J.C., Cole S.P. (2003). Identification of functional nuclear export sequences in human topoisomerase IIα and β. Biochem. Biophys. Res. Commun..

[99] Turner J.G., Engel R., Derderian J.A., Jove R., Sullivan D.M. (2004). Human topoisomerase IIα nuclear export is mediated by two CRM-1-dependent nuclear export signals. J. Cell Sci..

[100] Mirski S.E., Sparks K.E., Friedrich B., Kohler M., Mo Y.Y., Beck W.T., Cole S.P. (2007). Topoisomerase II binds importin α isoforms and exportin/CRM1 but does not shuttle between the nucleus and cytoplasm in proliferating cells. Exp. Cell Res..

[101] Arregi I., Falces J., Olazabal-Herrero A., Alonso-Marino M., Taneva S.G., Rodriguez J.A., Urbaneja M.A., Banuelos S. (2015). Leukemia-associated mutations in nucleophosmin alter recognition by CRM1: Molecular basis of aberrant transport. PLoS ONE.

[102] Ishida N., Hara T., Kamura T., Yoshida M., Nakayama K., Nakayama K.I. (2002). Phosphorylation of p27kip1 on serine 10 is required for its binding to CRM1 and nuclear export. J. Biol. Chem..

[103] Ishida N., Hara T., Kamura T., Yoshida M., Nakayama K., Nakayama K.I. (2015). Phosphorylation of p27kip1 on serine 10 is required for its binding to CRM1 and nuclear export. J. Biol. Chem..

[104] Connor M.K., Kotchetkov R., Cariou S., Resch A., Lupetti R., Beniston R.G., Melchior F., Hengst L., Slingerland J.M. (2003). CRM1/ran-mediated nuclear export of p27(kip1) involves a nuclear export signal and links p27 export and proteolysis. Mol. Biol. Cell.

[105] Zhang Y., Xiong Y. (2001). A p53 amino-terminal nuclear export signal inhibited by DNA damage-induced phosphorylation. Science.

[106] Santiago A., Li D., Zhao L.Y., Godsey A., Liao D. (2013). P53 sumoylation promotes its nuclear export by facilitating its release from the nuclear export receptor CRM1. Mol. Biol. Cell.

[107] Lohrum M.A., Woods D.B., Ludwig R.L., Balint E., Vousden K.H. (2001). *C*-terminal ubiquitination of p53 contributes to nuclear export. Mol. Cell Biol..

[108] Inoue T., Geyer R.K., Howard D., Yu Z.K., Maki C.G. (2001). MDM2 can promote the ubiquitination, nuclear export, and degradation of p53 in the absence of direct binding. J. Biol. Chem..

[109] Craig E., Zhang Z.K., Davies K.P., Kalpana G.V. (2002). A masked nes in INI1/HSNF5 mediates HCRM1-dependent nuclear export: Implications for tumorigenesis. EMBO J..

[110] Zhu J., McKeon F. (1999). NF-AT activation requires suppression of CRM1-dependent export by calcineurin (see comments). Nature.

[111] Jeyasekharan A.D., Liu Y., Hattori H., Pisupati V., Jonsdottir A.B., Rajendra E., Lee M., Sundaramoorthy E., Schlachter S., Kaminski C.F. (2013). A cancer-associated BRCA2 mutation reveals masked nuclear export signals controlling localization. Nat. Struct. Mol. Biol..

[112] Nitiss J.L. (2009). Targeting DNA topoisomerase II in cancer chemotherapy. Nat. Rev. Cancer.

[113] Nitiss J.L. (2009). DNA topoisomerase II and its growing repertoire of biological functions. Nat. Rev. Cancer.

[114] Wang J.C. (2002). Cellular roles of DNA topoisomerases: A molecular perspective. Nat. Rev. Mol. Cell Biol..

[115] Engel R., Valkov N.I., Gump J.L., Hazlehurst L., Dalton W.S., Sullivan D.M. (2004). The cytoplasmic trafficking of DNA topoisomerase IIα correlates with etoposide resistance in human myeloma cells. Exp. Cell Res..

[116] Valkov N.I., Sullivan D.M. (1997). Drug resistance to DNA topoisomerase I and II inhibitors in human leukemia, lymphoma, and multiple myeloma. Semin. Hematol..

[117] Turner J.G., Marchion D.C., Dawson J.L., Emmons M.F., Hazlehurst L.A., Washausen P., Sullivan D.M. (2009). Human multiple myeloma cells are sensitized to topoisomerase II inhibitors by CRM1 inhibition. Cancer Res..

[118] Turner J.G., Dawson J., Emmons M.F., Cubitt C.L., Kauffman M., Shacham S., Hazlehurst L.A., Sullivan D.M. (2013). CRM1 inhibition sensitizes drug resistant human myeloma cells to topoisomerase II and proteasome inhibitors both *in vitro* and *ex vivo*. J. Cancer.

[119] Jin Y., Xu X.L., Yang M.C., Wei F., Ayi T.C., Bowcock A.M., Baer R. (1997). Cell cycle-dependent colocalization of BARD1 and BRCA1 proteins in discrete nuclear domains. Proc. Natl. Acad. Sci. USA.

[120] Baer R., Ludwig T. (2002). The BRCA1/BARD1 heterodimer, a tumor suppressor complex with ubiquitin E3 ligase activity. Curr. Opin. Genet. Dev..

[121] Fabbro M., Rodriguez J.A., Baer R., Henderson B.R. (2002). BARD1 induces BRCA1 intranuclear foci formation by increasing ring-dependent BRCA1 nuclear import and inhibiting BRCA1 nuclear export. J. Biol. Chem..

[122] Scully R., Chen J., Ochs R.L., Keegan K., Hoekstra M., Feunteun J., Livingston D.M. (1997). Dynamic changes of BRCA1 subnuclear location and phosphorylation state are initiated by DNA damage. Cell.

[123] Rodriguez J.A., Schuchner S., Au W.W., Fabbro M., Henderson B.R. (2004). Nuclear-cytoplasmic shuttling of BARD1 contributes to its proapoptotic activity and is regulated by dimerization with BRCA1. Oncogene.

[124] Thompson M.E., Robinson-Benion C.L., Holt J.T. (2005). An amino-terminal motif functions as a second nuclear export sequence in BRCA1. J. Biol. Chem..

[125] Fabbro M., Henderson B.R. (2003). Regulation of tumor suppressors by nuclear-cytoplasmic shuttling. Exp. Cell Res..

[126] Rodriguez J.A., Span S.W., Ferreira C.G., Kruyt F.A., Giaccone G. (2002). CRM1-mediated nuclear export determines the cytoplasmic localization of the antiapoptotic protein survivin. Exp. Cell Res..

[127] Knauer S.K., Kramer O.H., Knosel T., Engels K., Rodel F., Kovacs A.F., Dietmaier W., Klein-Hitpass L., Habtemichael N., Schweitzer A. (2007). Nuclear export is essential for the tumor-promoting activity of survivin. FASEB J..

[128] Conway E.M., Pollefeyt S., Cornelissen J., DeBaere I., Steiner-Mosonyi M., Ong K., Baens M., Collen D., Schuh A.C. (2000). Three differentially expressed survivin cdna variants encode proteins with distinct antiapoptotic functions. Blood.

[129] Uren A.G., Wong L., Pakusch M., Fowler K.J., Burrows F.J., Vaux D.L., Choo K.H. (2000). Survivin and the inner centromere protein incenp show similar cell-cycle localization and gene knockout phenotype. Curr. Biol..

[130] Knauer S.K., Bier C., Habtemichael N., Stauber R.H. (2006). The survivin-CRM1 interaction is essential for chromosomal passenger complex localization and function. EMBO Rep..

[131] Polyak K., Lee M.H., Erdjument-Bromage H., Koff A., Roberts J.M., Tempst P., Massague J. (1994). Cloning of p27kip1, a cyclin-dependent kinase inhibitor and a potential mediator of extracellular antimitogenic signals. Cell.

[132] Rivard N., L’Allemain G., Bartek J., Pouyssegur J. (1996). Abrogation of p27kip1 by cdna antisense suppresses quiescence (G0 state) in fibroblasts. J. Biol. Chem..

[133] Coats S., Flanagan W.M., Nourse J., Roberts J.M. (1996). Requirement of p27kip1 for restriction point control of the fibroblast cell cycle. Science.

[134] Bouchard C., Thieke K., Maier A., Saffrich R., Hanley-Hyde J., Ansorge W., Reed S., Sicinski P., Bartek J., Eilers M. (1999). Direct induction of cyclin D2 by myc contributes to cell cycle progression and sequestration of p27. EMBO J..

[135] Perez-Roger I., Kim S.H., Griffiths B., Sewing A., Land H. (1999). Cyclins D1 and D2 mediate MYC-induced proliferation via sequestration of p27(kip1) and p21(cip1). EMBO J..

[136] Serres M.P., Zlotek-Zlotkiewicz E., Concha C., Gurian-West M., Daburon V., Roberts J.M., Besson A. (2011). Cytoplasmic p27 is oncogenic and cooperates with RAS both *in vivo* and *in vitro*. Oncogene.

[137] Sherr C.J., Roberts J.M. (1999). Cdk inhibitors: Positive and negative regulators of G1-phase progression. Genes Dev..

[138] Besson A., Assoian R.K., Roberts J.M. (2004). Regulation of the cytoskeleton: An oncogenic function for CDK inhibitors?. Nat. Rev. Cancer.

[139] Etchin J., Sanda T., Mansour M.R., Kentsis A., Montero J., Le B.T., Christie A.L., McCauley D., Rodig S.J., Kauffman M. (2013). Kpt-330 inhibitor of CRM1 (XPO1)-mediated nuclear export has selective anti-leukaemic activity in preclinical models of T-cell acute lymphoblastic leukaemia and acute myeloid leukaemia. Br. J. Haematol..

[140] Wang Y., Wang Y., Xiang J., Ji F., Deng Y., Tang C., Yang S., Xi Q., Liu R., Di W. (2014). Knockdown of CRM1 inhibits the nuclear export of p27(kip1) phosphorylated at serine 10 and plays a role in the pathogenesis of epithelial ovarian cancer. Cancer Lett..

[141] Noske A., Weichert W., Niesporek S., Roske A., Buckendahl A.C., Koch I., Sehouli J., Dietel M., Denkert C. (2008). Expression of the nuclear export protein chromosomal region maintenance/exportin 1/XPO1 is a prognostic factor in human ovarian cancer. Cancer.

[142] Huang W.Y., Yue L., Qiu W.S., Wang L.W., Zhou X.H., Sun Y.J. (2009). Prognostic value of CRM1 in pancreas cancer. Clin. Investig. Med..

[143] Azmi A.S., Aboukameel A., Bao B., Sarkar F.H., Philip P.A., Kauffman M., Shacham S., Mohammad R.M. (2013). Selective inhibitors of nuclear export block pancreatic cancer cell proliferation and reduce tumor growth in mice. Gastroenterology.

[144] Inoue H., Kauffman M., Shacham S., Landesman Y., Yang J., Evans C.P., Weiss R.H. (2012). CRM1 blockade by selective inhibitors of nuclear export (sine) attenuates kidney cancer growth. J. Urol..

[145] Van der Watt P.J., Maske C.P., Hendricks D.T., Parker M.I., Denny L., Govender D., Birrer M.J., Leaner V.D. (2009). The karyopherin proteins, CRM1 and karyopherin β1, are overexpressed in cervical cancer and are critical for cancer cell survival and proliferation. Intern. J. Cancer.

[146] Zhou F., Qiu W., Yao R., Xiang J., Sun X., Liu S., Lv J., Yue L. (2013). CRM1 is a novel independent prognostic factor for the poor prognosis of gastric carcinomas. Med. Oncol..

[147] Yao H. (2009). The expression of CRM1 is associated with prognosis in human osteosarcoma. Oncol. Rep..

[148] Shen A., Wang Y., Zhao Y., Zou L., Sun L., Cheng C. (2009). Expression of CRM1 in human gliomas and its significance in p27 expression and clinical prognosis. Neurosurgery.

[149] Lapalombella R., Sun Q., Williams K., Tangeman L., Jha S., Zhong Y., Goettl V., Mahoney E., Berglund C., Gupta S. (2012). Selective inhibitors of nuclear export show that CRM1/XPO1 is a target in chronic lymphocytic leukemia. Blood.

[150] Kojima K., Kornblau S.M., Ruvolo V., Dilip A., Duvvuri S., Davis R.E., Zhang M., Wang Z., Coombes K.R., Zhang N. (2013). Prognostic impact and targeting of CRM1 in acute myeloid leukemia. Blood.

[151] Zhang K., Wang M., Tamayo A.T., Shacham S., Kauffman M., Lee J., Zhang L., Ou Z., Li C., Sun L. (2013). Novel selective inhibitors of nuclear export CRM1 antagonists for therapy in mantle cell lymphoma. Exp. Hematol..

[152] Schmidt J., Braggio E., Kortuem K.M., Egan J.B., Zhu Y.X., Xin C.S., Tiedemann R.E., Palmer S.E., Garbitt V.M., McCauley D. (2013). Genome-wide studies in multiple myeloma identify XPO1/CRM1 as a critical target validated using the selective nuclear export inhibitor KPT-276. Leukemia.

[153] Tai Y.T., Landesman Y., Acharya C., Calle Y., Zhong M.Y., Cea M., Tannenbaum D., Cagnetta A., Reagan M., Munshi A.A. (2014). CRM1 inhibition induces tumor cell cytotoxicity and impairs osteoclastogenesis in multiple myeloma: Molecular mechanisms and therapeutic implications. Leukemia.

[154] Pathria G., Wagner C., Wagner S.N. (2012). Inhibition of CRM1-mediated nucleocytoplasmic transport: Triggering human melanoma cell apoptosis by perturbing multiple cellular pathways. J. Investig. Dermatol..

[155] Ranganathan P., Yu X., Na C., Santhanam R., Shacham S., Kauffman M., Walker A., Klisovic R., Blum W., Caligiuri M. (2012). Preclinical activity of a novel CRM1 inhibitor in acute myeloid leukemia. Blood.

[156] Etchin J., Sun Q., Kentsis A., Farmer A., Zhang Z.C., Sanda T., Mansour M.R., Barcelo C., McCauley D., Kauffman M. (2013). Antileukemic activity of nuclear export inhibitors that spare normal hematopoietic cells. Leukemia.

[157] Caceres-Gorriti K.Y., Carmona E., Barres V., Rahimi K., Letourneau I.J., Tonin P.N., Provencher D., Mes-Masson A.M. (2014). Ran nucleo-cytoplasmic transport and mitotic spindle assembly partners xpo7 and tpx2 are new prognostic biomarkers in serous epithelial ovarian cancer. PLoS ONE.

[158] Zhu J., Wang Y., Huang H., Yang Q., Cai J., Wang Q., Gu X., Xu P., Zhang S., Li M. (2015). Upregulation of kpnbeta1 in gastric cancer cell promotes tumor cell proliferation and predicts poor prognosis. Tumour Biol..

[159] Zhang P., Yang X., Ma X., Ingram D.R., Lazar A.J., Torres K.E., Pollock R.E. (2015). Antitumor effects of pharmacological EZH2 inhibition on malignant peripheral nerve sheath tumor through the miR-30a and KPNB1 pathway. Mol. Cancer.

[160] Zhang P., Garnett J., Creighton C.J., Al Sannaa G.A., Igram D.R., Lazar A., Liu X., Liu C., Pollock R.E. (2014). EZH2-miR-30d-KPNB1 pathway regulates malignant peripheral nerve sheath tumour cell survival and tumourigenesis. J. Pathol..

[161] Martens-de Kemp S.R., Nagel R., Stigter-van Walsum M., van der Meulen I.H., van Beusechem V.W., Braakhuis B.J., Brakenhoff R.H. (2013). Functional genetic screens identify genes essential for tumor cell survival in head and neck and lung cancer. Clin. Cancer Res..

[162] Kuusisto H.V., Jans D.A. (2015). Hyper-dependence of breast cancer cell types on the nuclear transporter importin β1. Biochim. Biophys. Acta.

[163] Christiansen A., Dyrskjot L. (2013). The functional role of the novel biomarker karyopherin α2 (KPNA2) in cancer. Cancer Lett..

[164] Fan H., Lu Y., Qin H., Zhou Y., Gu Y., Zhou J., Wang X., Fan D. (2013). High ran level is correlated with poor prognosis in patients with colorectal cancer. Int J. Clin. Oncol..

[165] Lu Y., Zhao X., Li K., Luo G., Nie Y., Shi Y., Zhou Y., Ren G., Feng B., Liu Z. (2013). Thioredoxin-like protein 2 is overexpressed in colon cancer and promotes cancer cell metastasis by interaction with ran. Antioxid. Redox Signal..

[166] Deng L., Lu Y., Zhao X., Sun Y., Shi Y., Fan H., Liu C., Zhou J., Nie Y., Wu K. (2013). Ran-GTPase protein promotes human pancreatic cancer proliferation by deregulating the expression of survivin and cell cycle proteins. Biochem. Biophys. Res. Commun..

[167] Chang K.C., Chang W.C., Chang Y., Hung L.Y., Lai C.H., Yeh Y.M., Chou Y.W., Chen C.H. (2013). Ran-GTPase-activating protein 1 is a therapeutic target in diffuse large B-cell lymphoma. PLoS ONE.

[168] Yuen H.F., Chan K.K., Grills C., Murray J.T., Platt-Higgins A., Eldin O.S., O’Byrne K., Janne P., Fennell D.A., Johnston P.G. (2012). Ran is a potential therapeutic target for cancer cells with molecular changes associated with activation of the PI3K/Akt/MTORC1 and Ras/MEK/ERK pathways. Clin. Cancer Res..

[169] Soderholm J.F., Bird S.L., Kalab P., Sampathkumar Y., Hasegawa K., Uehara-Bingen M., Weis K., Heald R. (2011). Importazole, a small molecule inhibitor of the transport receptor importin-β. ACS Chem. Biol..

[170] Kudo N., Khochbin S., Nishi K., Kitano K., Yanagida M., Yoshida M., Horinouchi S. (1997). Molecular cloning and cell cycle-dependent expression of mammalian CRM1, a protein involved in nuclear export of proteins. J. Biol. Chem..

[171] Murakami N., Ye Y., Kawanishi M., Aoki S., Kudo N., Yoshida M., Nakayama E.E., Shioda T., Kobayashi M. (2002). New rev-transport inhibitor with anti-HIV activity from valerianae radix. Bioorg. Med. Chem. Lett..

[172] Kudo N., Matsumori N., Taoka H., Fujiwara D., Schreiner E.P., Wolff B., Yoshida M., Horinouchi S. (1999). Leptomycin b inactivates CRM1/exportin 1 by covalent modification at a cysteine residue in the central conserved region. Proc. Natl. Acad. Sci. USA.

[173] Kudo N., Wolff B., Sekimoto T., Schreiner E.P., Yoneda Y., Yanagida M., Horinouchi S., Yoshida M. (1998). Leptomycin B inhibition of signal-mediated nuclear export by direct binding to CRM1. Exp. Cell Res..

[174] Hamamoto T., Gunji S., Tsuji H., Beppu T. (1983). Leptomycins a and b, new antifungal antibiotics. I. Taxonomy of the producing strain and their fermentation, purification and characterization. J. Antibiot. (Tokyo).

[175] Hamamoto T., Seto H., Beppu T. (1983). Leptomycins A and B, new antifungal antibiotics. II. Structure elucidation. J. Antibiot. (Tokyo).

[176] Hayakawa Y., Adachi K., Komeshima N. (1987). New antitumor antibiotics. J. Antibiot. (Tokyo).

[177] Hayakawa Y., Sohda K.Y., Shin-Ya K., Hidaka T., Seto H. (1995). Anguinomycins C and D, new antitumor antibiotics with selective cytotoxicity against transformed cells. J. Antibiot. (Tokyo).

[178] Bonazzi S., Eidam O., Guttinger S., Wach J.Y., Zemp I., Kutay U., Gademann K. (2010). Anguinomycins and derivatives: Total syntheses, modeling, and biological evaluation of the inhibition of nucleocytoplasmic transport. J. Am. Chem. Soc..

[179] Bonazzi S., Guttinger S., Zemp I., Kutay U., Gademann K. (2007). Total synthesis, configuration, and biological evaluation of anguinomycin C. Angew. Chem. Int. Ed..

[180] Schummer D., Gerth K., Reichenbach H., Hofle G. (1995). Antibiotics from gliding bacteria 63. Ratjadone—A new antifungal metabolite from sorangium-cellulosum. Liebigs Ann..

[181] Gerth K., Schummer D., Hofle G., Irschik H., Reichenbach H. (1995). Ratjadon: A new antifungal compound from sorangium cellulosum (myxobacteria) production, physio-chemical and biological properties. J. Antibiot. (Tokyo).

[182] Kalesse M., Christmann M., Bhatt U., Quitschalle M., Claus E., Saeed A., Burzlaff A., Kasper C., Haustedt L.O., Hofer E. (2001). The chemistry and biology of ratjadone. ChemBioChem.

[183] Williams D.R., Ihle D.C., Plummer S.V. (2001). Total synthesis of (−)-ratjadone. Org. Lett..

[184] Koster M., Lykke-Andersen S., Elnakady Y.A., Gerth K., Washausen P., Hofle G., Sasse F., Kjems J., Hauser H. (2003). Ratjadones inhibit nuclear export by blocking CRM1/exportin 1. Exp. Cell Res..

[185] Meissner T., Krause E., Vinkemeier U. (2004). Ratjadone and leptomycin B block CRM1-dependent nuclear export by identical mechanisms. FEBS Lett..

[186] Fleta-Soriano E., Martinez J.P., Hinkelmann B., Gerth K., Washausen P., Diez J., Frank R., Sasse F., Meyerhans A. (2014). The myxobacterial metabolite ratjadone a inhibits HIV infection by blocking the rev/CRM1-mediated nuclear export pathway. Microb. Cell Factories.

[187] Sun Q., Carrasco Y.P., Hu Y., Guo X., Mirzaei H., Macmillan J., Chook Y.M. (2013). Nuclear export inhibition through covalent conjugation and hydrolysis of leptomycin B by CRM1. Proc. Natl. Acad. Sci. USA.

[188] Roberts B.J., Hamelehle K.L., Sebolt J.S., Leopold W.R. (1986). *In vivo* and *in vitro* anticancer activity of the structurally novel and highly potent antibiotic Ci-940 and its hydroxy analog (pd 114,721). Cancer Chemother. Pharmacol..

[189] Newlands E.S., Rustin G.J., Brampton M.H. (1996). Phase i trial of elactocin. Br. J.Cancer.

[190] Mutka S.C., Yang W.Q., Dong S.D., Ward S.L., Craig D.A., Timmermans P.B., Murli S. (2009). Identification of nuclear export inhibitors with potent anticancer activity *in vivo*. Cancer Res..

[191] Daelemans D., Afonina E., Nilsson J., Werner G., Kjems J., De Clercq E., Pavlakis G.N., Vandamme A.M. (2002). A synthetic HIV-1 Rev inhibitor interfering with the CRM1-mediated nuclear export. Proc. Natl. Acad. Sci. USA.

[192] Van Neck T., Pannecouque C., Vanstreels E., Stevens M., Dehaen W., Daelemans D. (2008). Inhibition of the CRM1-mediated nucleocytoplasmic transport by *N*-azolylacrylates: Structure-activity relationship and mechanism of action. Bioorg. Med. Chem..

[193] Haines J.D., Herbin O., de la Hera B., Vidaurre O.G., Moy G.A., Sun Q., Fung H.Y., Albrecht S., Alexandropoulos K., McCauley D. (2015). Nuclear export inhibitors avert progression in preclinical models of inflammatory demyelination. Nat. Neurosci..

[194] Wang S., Han X., Wang J., Yao J., Shi Y. (2014). Antitumor effects of a novel chromosome region maintenance 1 (CRM1) inhibitor on non-small cell lung cancer cells *in vitro* and in mouse tumor xenografts. PLoS ONE.

[195] Walker C.J., Oaks J.J., Santhanam R., Neviani P., Harb J.G., Ferenchak G., Ellis J.J., Landesman Y., Eisfeld A.K., Gabrail N.Y. (2013). Preclinical and clinical efficacy of XPO1/CRM1 inhibition by the karyopherin inhibitor KPT-330 in pH^+^ leukemias. Blood.

[196] London C.A., Bernabe L.F., Barnard S., Kisseberth W.C., Borgatti A., Henson M., Wilson H., Jensen K., Ito D., Modiano J.F. (2014). Preclinical evaluation of the novel, orally bioavailable selective inhibitor of nuclear export (sine) KPT-335 in spontaneous canine cancer: Results of a phase I study. PLoS ONE.

[197] Hilliard M., Frohnert C., Spillner C., Marcone S., Nath A., Lampe T., Fitzgerald D.J., Kehlenbach R.H. (2010). The anti-inflammatory prostaglandin 15-deoxy-δ(12,14)-pgj2 inhibits CRM1-dependent nuclear protein export. J. Biol. Chem..

[198] Niu M., Wu S., Mao L., Yang Y. (2013). CRM1 is a cellular target of curcumin: New insights for the myriad of biological effects of an ancient spice. Traffic.

[199] Aggarwal B.B., Kumar A., Bharti A.C. (2003). Anticancer potential of curcumin: Preclinical and clinical studies. Anticancer Res..

[200] Sharma R.A., Euden S.A., Platton S.L., Cooke D.N., Shafayat A., Hewitt H.R., Marczylo T.H., Morgan B., Hemingway D., Plummer S.M. (2004). Phase I clinical trial of oral curcumin: Biomarkers of systemic activity and compliance. Clin. Cancer Res..

[201] Silva A.L., Rech S.B., von Poser G.L. (2002). Quantitative determination of valepotriates from valeriana native to south brazil. Planta Medica.

[202] Tamura S., Shimizu N., Fujiwara K., Kaneko M., Kimura T., Murakami N. (2010). Bioisostere of valtrate, anti-HIV principle by inhibition for nuclear export of rev. Bioorg. Med. Chem. Lett..

[203] Liu X., Niu M., Xu X., Cai W., Zeng L., Zhou X., Yu R., Xu K. (2014). CRM1 is a direct cellular target of the natural anti-cancer agent plumbagin. J. Pharmacol. Sci..

[204] Niu M., Xu X., Shen Y., Yao Y., Qiao J., Zhu F., Zeng L., Liu X., Xu K. (2015). Piperlongumine is a novel nuclear export inhibitor with potent anticancer activity. Chem. Biol. Interact..

[205] Chen W.Y., Wu C.C., Lan Y.H., Chang F.R., Teng C.M., Wu Y.C. (2005). Goniothalamin induces cell cycle-specific apoptosis by modulating the redox status in MDA-MB-231 cells. Eur. J. Pharmacol..

[206] Wach J.Y., Guttinger S., Kutay U., Gademann K. (2010). The cytotoxic styryl lactone goniothalamin is an inhibitor of nucleocytoplasmic transport. Bioorg. Med. Chem. Lett..

[207] De Fatima A., Kohn L.K., Antonio M.A., de Carvalho J.E., Pilli R.A. (2005). (*R*)-goniothalamin: Total syntheses and cytotoxic activity against cancer cell lines. Bioorg. Med. Chem..

[208] Al-Qubaisi M., Rozita R., Yeap S.K., Omar A.R., Ali A.M., Alitheen N.B. (2011). Selective cytotoxicity of goniothalamin against hepatoblastoma HEPG2 cells. Molecules.

[209] Chiu C.C., Liu P.L., Huang K.J., Wang H.M., Chang K.F., Chou C.K., Chang F.R., Chong I.W., Fang K., Chen J.S. (2011). Goniothalamin inhibits growth of human lung cancer cells through DNA damage, apoptosis, and reduced migration ability. J. Agric. Food Chem..

[210] Ye Y., Li B. (2006). 1ʹs-1ʹ-acetoxychavicol acetate isolated from alpinia galanga inhibits human immunodeficiency virus type 1 replication by blocking rev transport. J. Gen. Virol..

[211] Tamura S., Shiomi A., Kaneko M., Ye Y., Yoshida M., Yoshikawa M., Kimura T., Kobayashi M., Murakami N. (2009). New rev-export inhibitor from alpinia galanga and structure-activity relationship. Bioorg. Med. Chem. Lett..

[212] Murakami N., Sugimoto M., Kobayashi M. (2001). Participation of the β-hydroxyketone part for potent cytotoxicity of callystatin a, a spongean polyketide. Bioorg. Med. Chem..

[213] Kalesse M., Chary K.P., Quitschalle M., Burzlaff A., Kasper C., Scheper T. (2003). The total synthesis of (−)-callystatin a. Chemistry.

[214] Langille N.F., Panek J.S. (2004). Total synthesis of (−)-callystatin a. Org. Lett..

[215] Hayakawa Y., Sohda K., Furihata K., Kuzuyama T., Shin-ya K., Seto H. (1996). Studies on new antitumor antibiotics, leptofuranins A, B, C and D.I. Taxonomy, fermentation, isolation and biological activities. J. Antibiot. (Tokyo).

[216] Hayakawa Y., Sohda K., Seto H. (1996). Studies on new antitumor antibiotics, leptofuranins A, B, C and D II. Physiocochemical properties and structure elucidation. J. Antibiot. (Tokyo).

[217] Sakakibara K., Saito N., Sato T., Suzuki A., Hasegawa Y., Friedman J.M., Kufe D.W., Vonhoff D.D., Iwami T., Kawabe T. (2011). Cbs9106 is a novel reversible oral CRM1 inhibitor with CRM1 degrading activity. Blood.

[218] Almholt D.L., Loechel F., Nielsen S.J., Krog-Jensen C., Terry R., Bjorn S.P., Pedersen H.C., Praestegaard M., Moller S., Heide M. (2004). Nuclear export inhibitors and kinase inhibitors identified using a mapk-activated protein kinase 2 redistribution screen. Assay Drug Dev. Technol..

[219] Kau T.R., Schroeder F., Ramaswamy S., Wojciechowski C.L., Zhao J.J., Roberts T.M., Clardy J., Sellers W.R., Silver P.A. (2003). A chemical genetic screen identifies inhibitors of regulated nuclear export of a forkhead transcription factor in pten-deficient tumor cells. Cancer Cell.

[220] Salas Fragomeni R.A., Chung H.W., Landesman Y., Senapedis W., Saint-Martin J.R., Tsao H., Flaherty K.T., Shacham S., Kauffman M., Cusack J.C. (2013). CRM1 and braf inhibition synergize and induce tumor regression in braf-mutant melanoma. Mol. Cancer Ther..

[221] Abraham S.A., Holyoake T.L. (2013). Redirecting traffic using the XPO1 police. Blood.

[222] Wettersten H.I., Landesman Y., Friedlander S., Shacham S., Kauffman M., Weiss R.H. (2014). Specific inhibition of the nuclear exporter exportin-1 attenuates kidney cancer growth. PLoS ONE.

[223] De Cesare M., Cominetti D., Doldi V., Lopergolo A., Deraco M., Gandellini P., Friedlander S., Landesman Y., Kauffman M.G., Shacham S. (2015). Anti-tumor activity of selective inhibitors of XPO1/CRM1-mediated nuclear export in diffuse malignant peritoneal mesothelioma: The role of survivin. Oncotarget.

[224] Niu M., Chong Y., Han Y., Liu X. (2015). Novel reversible selective inhibitor of nuclear export shows that CRM1 is a target in colorectal cancer cells. Cancer Biol. Ther..

[225] Senapedis W.T., Baloglu E., Landesman Y. (2014). Clinical translation of nuclear export inhibitors in cancer. Semin. Cancer Biol..

[226] Das A., Wei G., Parikh K., Liu D. (2015). Selective inhibitors of nuclear export (sine) in hematological malignancies. Exp. Hematol. Oncol..

[227] Ishizawa J., Kojima K., Hail N., Tabe Y., Andreeff M. (2015). Expression, function, and targeting of the nuclear exporter chromosome region maintenance 1 (CRM1) protein. Pharmacol. Ther..

[228] Parikh K., Cang S., Sekhri A., Liu D. (2014). Selective inhibitors of nuclear export (sine)—A novel class of anti-cancer agents. J. Hematol. Oncol..

[229] Kazim S., Malafa M.P., Coppola D., Husain K., Zibadi S., Kashyap T., Crochiere M., Landesman Y., Rashal T., Sullivan D.M. (2015). Selective nuclear export inhibitor KPT-330 enhances the antitumor activity of gemcitabine in human pancreatic cancer. Mol. Cancer Ther..

[230] Hing Z.A., Mantel R., Beckwith K.A., Guinn D., Williams E., Smith L.L., Williams K., Johnson A.J., Lehman A.M., Byrd J.C. (2015). Selinexor is effective in acquired resistance to ibrutinib and synergizes with ibrutinib in chronic lymphocytic leukemia. Blood.

[231] Bernad R., van der Velde H., Fornerod M., Pickersgill H. (2004). Nup358/ RanBP2 attaches to the nuclear pore complex via association with Nup88 and Nup214/can and plays a supporting role in CRM1-mediated nuclear protein export. Mol. Cell Biol..

[232] Hutten S., Kehlenbach R.H. (2006). Nup214 is required for CRM1-dependent nuclear protein export *in vivo*. Mol. Cell Biol..

[233] Roth P., Xylourgidis N., Sabri N., Uv A., Fornerod M., Samakovlis C. (2003). The drosophila nucleoporin DNUP88 localizes DNUP214 and CRM1 on the nuclear envelope and attenuates nes-mediated nuclear export. J. Cell Biol..

[234] Zhao C.L., Mahboobi S.H., Moussavi-Baygi R., Mofrad M.R. (2014). The interaction of CRM1 and the nuclear pore protein tpr. PLoS ONE.

[235] Waldmann I., Spillner C., Kehlenbach R.H. (2012). The nucleoporin-like protein NLP1 (HCG1) promotes CRM1-dependent nuclear protein export. J. Cell Sci..

[236] Takeda A., Sarma N.J., Abdul-Nabi A.M., Yaseen N.R. (2010). Inhibition of CRM1-mediated nuclear export of transcription factors by leukemogenic Nup98 fusion proteins. J. Biol. Chem..

[237] Roloff S., Spillner C., Kehlenbach R.H. (2013). Several phenylalanine-glycine motives in the nucleoporin Nup214 are essential for binding of the nuclear export receptor CRM1. J. Biol. Chem..

[238] Oka M., Asally M., Yasuda Y., Ogawa Y., Tachibana T., Yoneda Y. (2010). The mobile fg nucleoporin NUP98 is a cofactor for CRM1-dependent protein export. Mol. Biol. Cell.

[239] Bernad R., Engelsma D., Sanderson H., Pickersgill H., Fornerod M. (2006). Nup214- Nup88 nucleoporin subcomplex is required for CRM1-mediated 60 s preribosomal nuclear export. J. Biol. Chem..

